# Unveiling the factors influencing public knowledge and behaviours towards medication errors in Jordan: a cross-sectional study

**DOI:** 10.1186/s12913-024-11230-6

**Published:** 2024-07-10

**Authors:** Sura Al Zoubi, Lobna Gharaibeh, Enas A. Amaireh, Husam AlSalamat, Mohammad Ghassab Deameh, Amjad Almansi, Yaqeen Majed Al Asoufi, Hadeel Alshahwan, Zaha Al-Zoubi

**Affiliations:** 1 Department of Basic Medical Sciences, Faculty of Medicine, Al-Balqa Applied University, As-Salt, Jordan; 2 Pharmacological and Diagnostic Research Center, Biopharmaceutics and Clinical Pharmacy Department, Faculty of Pharmacy, Al-Ahliyya Amman University, Amman, Jordan; 3 The Speciality Hospital, Amman, Jordan; 4 Prince Hamza Hospital, Amman, Jordan; 5 Jordanian Royal Medical Services, Amman, Jordan; 6 Independent Researcher, Amman, Jordan

**Keywords:** Medication errors, Patient safety, Patient engagement, Medication safety, Medication use

## Abstract

**Background:**

Medication errors are preventable incidents resulting from improper use of drugs that may cause harm to patients. They thus endanger patient safety and offer a challenge to the efficiency and efficacy of the healthcare system. Both healthcare professionals and patients may commit medication errors.

**Methods and objectives:**

A cross-sectional, observational study was designed using a self-developed, self-administered online questionnaire. A sample was collected using convenience sampling followed by snowball sampling. Adult participants from the general population were recruited regardless of age, gender, area of residence, medical history, or educational background in order to explore their practice, experience, knowledge, and fear of medication error, and their understanding of this drug-related problem.

**Results:**

Of the 764 participants who agreed to complete the questionnaire, 511 (66.9%) were females and 295 (38.6%) had a medical background. One-fifth of participants had experienced medication errors, with 37.7% of this segment reporting these medication errors. More than half of all medication errors (84, 57.5%) were minor and thus did not require any intervention. The average anxiety score for all attributes was 21.2 (The highest possible mean was 36, and the lowest possible was 0). The highest level of anxiety was seen regarding the risk of experiencing drug-drug interactions and the lowest levels were around drug costs and shortages. Being female, having no medical background, and having experience with medication errors were the main predictors of high anxiety scores. Most participants (between 67% and 92%) were able to recognise medication errors committed by doctors or pharmacists. However, only 21.2 to 27.5% of participants could recognise medication errors committed by patients. Having a medical background was the strongest predictor of knowledge in this study (*P* < 0.001).

**Conclusion:**

The study revealed that the prevalence of self-reported medication errors was significantly high in Jordan, some of which resulted in serious outcomes such as lasting impairment, though most were minor. Raising awareness about medication errors and implementing preventive measures is thus critical, and further collaboration between healthcare providers and policymakers is essential to educate patients and establish effective safety protocols.

**Supplementary Information:**

The online version contains supplementary material available at 10.1186/s12913-024-11230-6.

## Introduction

Patient safety is an absolute priority in the healthcare industry and a major attribute of a high-quality healthcare system [1]. Preserving an error-free environment while providing effective patient care is the ultimate goal of the patient care process [2]. However, medical errors still occur frequently, with medical errors being ranked as the third most common cause of death in 2016 in the United States [3]. Medication errors (MEs) are the most common type of medical errors, which pose a significant challenge to patient safety and the overall efficacy of the healthcare system [4]. MEs are also recognised as the primary cause of preventable patient harm within the global healthcare system [5, 6]. Such errors can arise at any stage of the medication administration process [7], including during prescribing, dispensing, administration, and monitoring [8], though the highest prevalence of errors occurs in the prescribing and monitoring stages [9]. Various strategies and approaches that involve different stakeholders can improve patient safety and reduce medication errors [10–12].

Medication errors may occur in both inpatient and outpatient settings [13] and in different patient population [14]. Various individuals, including any healthcare personnel, may be responsible for medication errors, including doctors, pharmacists, the patients themselves, their caregivers, or their families [15–17]. The prevalence of medication errors among various personnel varies between studies [18–21], and while healthcare providers and patients are encouraged to report all medication errors encountered to the Food and Drug Administration (FDA) [22], medication errors remain underreported [23, 24].

The causes of medication errors are multifactorial, with such factors including staff shortages, misinterpretation of prescriptions or medication charts, inadequate knowledge, and limited experience [25]. However, it is important to note that MEs most commonly originate from inadequately designed work environments and systems rather than hinging on the individual performance of a single healthcare practitioner [26].

Medication errors are preventable and as such may lead to avoidable harm. Although not all MEs are inherently harmful to patients [27, 28], some MEs result in substantial adverse outcomes including prolonged hospital stays, increased healthcare costs, complications related to medication, and potential harm to patients, potentially including death [29, 30]. Additionally, such errors have the potential to significantly undermine public confidence and trust in medical services [31] as well as to expose healthcare personnel to legal consequences such as being sued for medical malpractice [13].

Various studies, including those in Jordan, have studied the prevalence, causes and rate of MEs in different healthcare settings and practices [9, 19, 32, 33]. Other studies have described a range of knowledge, beliefs, and attitudes regarding medication safety and medication errors from the perspective of healthcare professionals [17, 34, 35]. Patients’ role in the safe use of medication is also crucial, and patients must be encouraged to improve their own medication safety [36]. However, studies examining patient engagement in error prevention and the medication errors committed by patients remain scanty [8], while studies about the general population’s knowledge and beliefs about medication errors are significantly lacking.

Within the existing literature on MEs nationally and internationally, there is thus a noticeable gap in terms of understanding the perspectives of the patients and the general population regarding MEs. To the best of the authors’ knowledge, no studies in Jordan have explored the general population’s viewpoints, knowledge, and fear of medication errors or their understanding of drug-related problems. This study aims to fill this gap and to shed light on potential key factors related to MEs, thereby contributing valuable insights to the existing literature.

## Methodology

### Research design and participants

For this research, a cross-sectional study was designed. An online questionnaire was developed by the researchers for this study based on a careful review of the existing literature. Some questions were adopted, with slight modifications, from similar research and other questions were formulated by the researchers to cover the aims of this research. The target for this study was the adult population in Jordan. Individuals were approached to fill out the questionnaire regardless of age as long as they were aged 18 years or older, and without regard to area of residence, gender, educational background, or medical history. In order to get a high response rate for this online questionnaire, convenience sampling followed by snowball sampling was used. A target of a minimum of 385 participants was set after calculation of the required sample size using an online sampling calculator (Raosoft^®^) [37] set to the following values: a confidence level of 95%, a margin of error of 5% and a null response distribution of 50%, taking into consideration the Jordanian population of approximately 11.3 million of whom approximately 6.4 million were aged over 18 in 2023 [38]. However, a much bigger sample was collected (764 participants), increasing the power of this study.

### Survey procedures and tool

Once ethical approval was granted by the Research Ethics Committee of the School of Medicine at Al-Balqa Applied University BAU and the Institutional Review Board (IRB) at Al-Balqa Applied University (Ref no. 1762/1/3/26), the face and content validity of the questionnaire were tested. To evaluate face validity, a group from the target population were asked to review the questionnaire for its suitability, extensiveness and clarity. For content validity, a team of experts from the field (including a pharmacist, pharmacologist, practising physician, and public health expert) were asked to assess how well the measure in each question was constructed. Amendments were made as needed, and the final version was then retested for content validity before being used to conduct a pilot study on 25 participants. The data from this pilot was excluded from the final analysis. The questionnaire was presented in the Arabic language and an accompanying Google form was created with a cover letter that provided details about the study’s purpose, the time required to fill out the questionnaire, the voluntary nature of participation, the participants’ rights to withdraw at any point, and a confidentiality statement. There was also a statement that read: “Participation in this study is voluntary and by completing and submitting this form, participants are providing us with consent to use their data for this study.”. However, the definition of medication errors was not provided as the main purpose of this study was to measure participants’ knowledge about medication errors including its definition and providing the definition would conflict with this purpose. The link to the questionnaire was distributed via emails and various social media platforms, and the instrument was opened up to responses throughout October and November 2023.

The questionnaire (Supplementary table S1) contained 17 questions that were divided into four sections concerning the participants’ sociodemographic characteristics (seven questions), medication safety behaviour and experience concerning medication errors (seven questions), participants’ beliefs about the healthcare system and anxiety around medication errors (one question with nine scenarios to rate), and, finally, a section about their knowledge about different types of medication errors (one question with 12 cases to categorise) and their prevention and consequences (one question with five sentences to assign frequencies for). The 2017 WHO definition of medication error [39] was used as a reference to measure the participants’ knowledge scores.

### Statistical analysis

The data was analysed using IBM SPSS Version 25.0. Descriptive analysis, including frequencies and percentages, was used for categorical variables, while means and standard deviations (SD) were used to describe the continuous variables. The reliability of the attributes of anxiety and types of knowledge was checked using Cronbach’s alpha with the following results 0.91 and 0.60, respectively. An acceptable value of Cronbach’s alpha is ≥ 0.6 [40].

Pearson correlation was utilised to detect correlations between two continuous variables. The strength of correlation increases as the Pearson correlation approaches ± 1, and zero indicates no correlation [41]. Univariate and multivariate linear regression were used to evaluate possible predictors of adequate knowledge and anxiety. All variables with *P* values < 0.25 in the univariate analysis were introduced to the multivariate linear regression. Beta regression coefficient indicates the direction and effect size, larger values indicate stronger effect. *P* values ≤ 0.05 were considered statistically significant.

## Results

A total of 764 participants agreed to fill out the questionnaire, more than half were females (511, 66.9%). The mean age was 31.4 ± 12.0 years, only 38.6% had a medical background, 41.4% used drugs regularly, and more than three-quarters (591, 77.4%) had medical insurance, Table 1.

**Table 1:** Sociodemographic characteristics of the participants, *N* = 764

	*N*	%
**Gender**		
Male	253	33.1
Female	511	66.9
**Education level**		
School level	36	4.7
Diploma/community college	48	6.3
University level	580	75.9
Postgraduate level	100	13.1
**Do you have a medical background?**		
No	469	61.4
Yes	295	38.6
**Do you use drugs regularly?**		
No	448	58.6
Yes	316	41.4
**Allergies**		
No	703	92.0
Yes	61	8.0
**Medical insurance**		
No	173	22.6
Yes	591	77.4

Evaluation of the practices of the participants revealed that most of them depended on their memory to remember taking their medication, rarely kept their medication in a pharmacy cabinet, and (291, 38.1%) used to forget to take their medication at least sometimes. One-fifth of the participants experienced medication errors and 37.7% of them reported these medication errors. More than half of these medication errors (84, 57.5%) were minor and did not need any intervention Table 2.

**Table 2:** Participants´ medication safety behaviour and experiences concerning medication error, *N* = 764

	*N*	%
**How do you remember taking your drugs?**		
I depend on my memory	581	76.0
Someone else reminds me (a family member or a friend)	65	8.5
I use phone apps	105	13.7
Other*	13	1.8
**How often do you forget to take your medicine?**		
Never	86	11.3
Rarely	387	50.7
Sometimes	260	34.0
Always	31	4.1
**Where do you keep your medication?**		
Pharmacy cabinet	42	5.5
Kitchen	278	36.4
Bathroom	4	0.5
Bedroom	316	41.4
Office	45	5.9
Refrigerator	2	0.3
Car	2	0.3
Living room	66	8.6
Other†	9	1.2
**Has a family member or friend experienced medication errors?**		
No	541	70.8
Yes	223	29.2
**Have you ever experienced a medication error?**		
No	618	80.9
Yes	146	19.1
**Did you report the medication error? (** ***N*** ** = 146, participants who experience medication error)**		
No	91	62.3
Yes	55	37.7
**Severity of medication error (** ***N*** ** = 146, participants who experience medication error)**		
Medication errors that caused permanent disability	6	4.1
Major medication errors that needed medical intervention from the physician or pharmacist	9	6.2
Moderate medication error that needed intervention from the physician or pharmacist	46	31.5
Minor medication error that did not need any intervention	85	58.2

*: I receive a message from the pharmacy, I write notes around the house, I remember when it is prayer time, or I remember when I see the drug in front of me

†: Participants did not specify

**Fig. 1:**
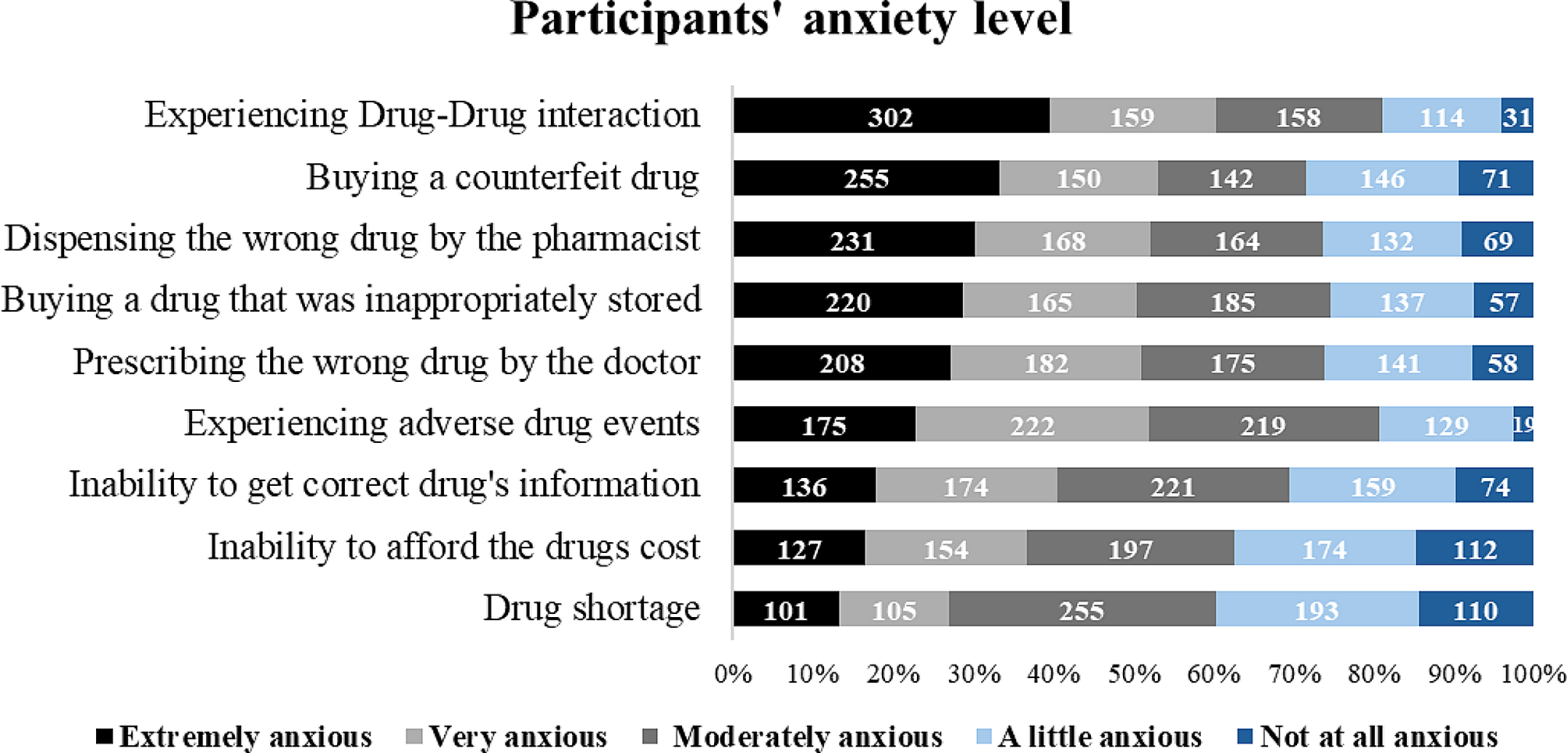
Participants’ anxiety level. Participants (*N* = 764) were asked to rank their anxiety to potential medication errors

The anxiety levels of each of the anxiety attributes are shown in Fig. 1. The highest levels of anxiety were regarding experiencing drug-drug interaction, buying counterfeit drugs or dispensing the wrong drug by the pharmacist. The lowest levels were about the inability to afford to buy the drugs or facing unavailability of drugs.

The average anxiety score for all attributes was 21.2 (The highest possible mean is 36, and the lowest possible mean is 0). The total score for each participant was summed in a total score, high scores reflected a high degree of anxiety. Predictors of anxiety score are assessed in Table 3. In the multivariate analysis, females were more anxious compared to males, participants who experienced medication errors were more anxious compared to those who did not have such experience, and participants with medical backgrounds were less anxious compared to others.

**Table 3:** Analysis of possible predictors of anxiety score using linear regression, *N* = 764

	Univariate linear regression			Multivariate linear regression		
	B	95% CI	*P*	B	95% CI	*P*
**Age**	0.072	0.020–0.125	0.007^Ω^	0.023	-0.034-0.081	0.425
**Gender**						
Male ^€^						
Female	1.964	0.622–3.305	0.004 ^Ω^	1.698	0.354–3.042	0.013
**Education level**						
School level ^€^						
Diploma/community college	-1.694	-5.560-2.171	0.390			
University level	-2.316	-5.327-0.696	0.132			
Postgraduate level	-1.458	-4.866-1.950	0.401			
**Do you have a medical background?**						
No ^€^						
Yes	-3.168	-4.452- -1.884	< 0.001 ^Ω^	-2.792	-4.179-1.406	< 0.001
**Do you use drugs regularly?**						
No ^€^						
Yes	0.927	-0.361-2.214	0.158 ^Ω^	-0.059	-1.385-1.266	0.93
**Allergies**						
No ^€^						
Yes	3.393	1.063–5.722	0.004 ^Ω^	2.317	-0.081-4.716	0.058
**Medical insurance**						
No ^€^						
Yes	-0.631	-2.147-0.884	0.414			
**Have you ever experienced a medication error?**						
No ^€^						
Yes	2.832	1.231–4.434	0.001 ^Ω^	2.358	0.677–4.039	0.006
**has a family member or friend experienced medication errors?**						
No ^€^						
Yes	0.83	-0.557-2.233	0.238 ^Ω^	0.168	-1.243-1.580	0.815

*B*: Regression coefficient; 95% CI: 95% Confidence interval. Variables with a *P* value < 0.25 in the univariate linear model were analysed in the multivariate linear regression; ^€^: Reference

**Fig. 2:**
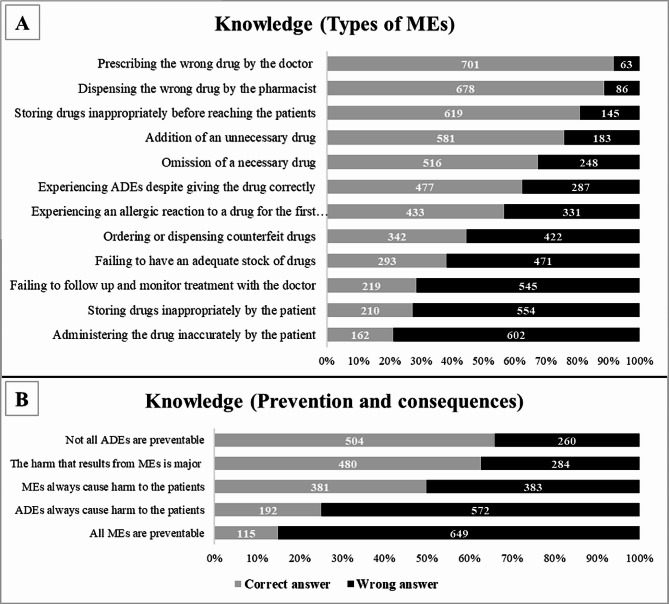
Participants’ knowledge. Participants (*N* = 764) were asked to (**A**) identify medication errors from several scenarios and (**B**) give frequencies about the prevention and consequences of medication errors

The frequency and percentage of correct answers for every knowledge attribute (different types of medication error) were calculated and presented in Fig. 2. The highest percentages for correct answers were “Prescribing the wrong drug by the doctor” (91.8%) and “Dispensing the wrong drug by the pharmacist” (88.7%). The lowest were “Failing to follow up and monitor treatment with the doctor”, “Storing drugs inappropriately by the patient” and “Administering the drug inaccurately by the patient” (28.7%, 27.5% and 21.2% respectively).

In terms of knowledge of the prevention and consequences of medication error, the highest percentage for correct answers was “Not all ADEs are preventable” (65.9%), and the lowest was “All MEs are preventable” (15.0%), Fig. 2.

The knowledge scores were summed for every participant and the possible predictors were assessed using linear regression, Table 4. The only variable that predicted knowledge of different types of medication errors was having a medical background. Participants with a medical background had better knowledge compared to those without any medical background.

**Table 4:** Possible predictors of knowledge scores concerning different types of medication error using linear regression analysis, *N* = 764

	Univariate linear regression			Multivariate linear regression		
	B	95% CI	*P*	B	95% CI	*P*
**Age**	-0.011	-0.024-0.002	0.099	-0.002	-0.016-0.013	0.824
**Gender**						
Male ^€^						
Female	-0.172	-0.506-0.163	0.314			
**Education level**						
School level ^€^						
Diploma/community college	0.243	-0.717-1.203	0.619			
University level	0.379	-0.369-1.127	0.320			
Postgraduate level	0.052	-0.794-0.898	0.904			
**Do you have a medical background?**						
No ^€^						
Yes	0.612	0.292–0.933	< 0.001	0.598	0.250–0.945	0.001
**Do you use drugs regularly?**						
No ^€^						
Yes	-0.047	-0.367-0.273	0.773			
**Allergies**						
No ^€^						
Yes	-0.216	-0.797-0.365	0.467			
**Medical insurance**						
No ^€^						
Yes	0.111	-0.265-0.488	0.561			
**Have you ever experienced a medication error?**						
No ^€^						
Yes	-0.214	-0.615-0.186	0.294	-0.217	-0.614-0.181	0.285
**has a family member or friend experienced medication errors?**						
No ^€^						
Yes	0.109	-0.238-0.455	0.537			

*B*: Regression coefficient; 95% CI: 95% Confidence interval. Variables with a *P* value < 0.25 in the univariate linear model were analysed in the multivariate linear regression; ^€^: Reference

Gender and having a medical background were significant predictors of knowledge scores concerning the prevention and consequences of medication error. Females and those with medical knowledge had higher knowledge scores than males and participants without medical background, Table 5.

**Table 5:** Possible predictors of knowledge scores concerning prevention and consequences of medication error using linear regression analysis

	Univariate linear regression			Multivariate linear regression		
	B	95% CI	*P*	B	95% CI	*P*
**Age**	-0.006	-0.012-0.000	0.066	-0.001	-0.008-0.006	0.834
**Gender**						
Male ^€^						
Female	-0.185	-0.337- -0.033	0.017	-0.151	-0.303-0.000	0.05
**Education level**						
School level ^€^						
Diploma/community college	0.264	-0.174-0.701	0.237	0.235	-0.200-0.670	0.290
University level	0.278	-0.063-0.619	0.110	0.126	-0.218-0.470	0.471
Postgraduate level	0.126	-0.260-0.511	0.523	0.041	-0.346-0.428	0.836
**Do you have a medical background?**						
No ^€^						
Yes	0.345	0.199–0.490	< 0.001	0.306	0.146–0.466	< 0.001
**Do you use drugs regularly?**						
No ^€^						
Yes	-0.030	-0.176-0.116	0.687			
**Allergies**						
No ^€^						
Yes	0.098	0-0.167-0.363	0.468			
**Medical insurance**						
No ^€^						
Yes	0.191	0.020–0.363	0.029	0.169	-0.004-0.342	0.056
**Have you ever experienced a medication error?**						
No ^€^						
Yes	0.055	-0.128-0.238	0.556			
**has a family member or friend experienced medication errors?**						
No ^€^						
Yes	0.031	-0.127-0.190	0.696			

*B*: Regression coefficient; 95% CI: 95% Confidence interval. Variables with a *P* value < 0.25 in the univariate linear model were analysed in the multivariate linear regression; ^€^: Reference

There was no significant correlation between the knowledge score of different types of medication error and the anxiety score, Pearson correlation of -0.028, *P* = 0.447. However, there was a significant, but weak, negative correlation between the knowledge score concerning the prevention and consequences of medication error and the anxiety score, Pearson correlation of -0.087, *P* = 0.016.

Participants who had low knowledge scores regarding different types of medication error had lower knowledge scores regarding the prevention and consequences of medication errors, Pearson correlation of 0.178, *P* value < 0.001.

## Discussion

This is the first study in Jordan to describe medication errors from a patient perspective. Participants from a range of different sociodemographic backgrounds were thus approached to take part in this study regardless of their educational backgrounds or medical history. Of the 764 participants, fifth had experienced a medication error themselves and almost third knew a family member or a friend who had. Similarly, a study that was conducted at the University of Michigan showed that 18.3% and 21.0% of participants had personal experience of, or knew someone who had experienced, medication errors, respectively [8]. Another research that reported on medical errors more generally showed that 21% of Americans had individual experience with medical errors and 31% cared for someone who had had such an experience [42]. More than half of the participants who had experienced medication errors in this study ranked these as minor errors that had neither caused any harm nor required any intervention. However, 10.3% of participants had experienced major medication errors that had necessitated medical intervention (6.2%) or led to permanent disability (4.1%). A higher percentage (22%) of serious medication errors was reported by The Commonwealth Fund 2001 Health Care Quality Survey [43]. However, much has been done since that date to lower the incidence of medication errors that may cause severe harm to patients, which may well have improved patient safety [39]. Only 37.7% of the participants who experienced medication errors in this study reported these to their healthcare provider, however, and studies usually measure the reporting of medication errors by healthcare professionals, not by the patients themselves. Overall, studies suggest that medication errors are generally underreported, however. The main reason for this may be a fear of consequences and the associated legal implications among healthcare providers [23, 44]. However, while this should not be a concern for patients, although patients are encouraged to report any medication errors they encounter, they still do not do this regularly. Some patients are worried about the doctors’ response to such reports, while others think that nothing will change even if they make such reports [42, 45].

Participants in the current study showed various levels of anxiety about different potential errors that they might face. They were most anxious about experiencing drug-drug interactions, buying adulterated drugs, or getting the wrong drugs from the pharmacy. These results may be expected, as prescribing and dispensing errors are the most common types of medication errors [9]. Additionally, they align with the results from a survey conducted on more than a thousand adults by the American Society of Health-System Pharmacists [46], in which more than half of participants were very concerned about being given the wrong drug or receiving drugs that might interact with each other. In the same survey, 58% of participants showed significant concern about the cost of treatment, however, while in the current study, participants were least anxious about their inability to afford drug costs. This might be because most participants in the current work had health insurance that covers most, if not all, of their medical expenses. As per the Household Expenditure and Income Survey conducted in Jordan in 2017, most Jordanians have some kind of health insurance for such purposes [47].

The current study revealed that females are significantly more anxious about medication errors than males (*P* < 0.05), which is unsurprising, as global research shows that, compared to males, the likelihood of anxiety disorders in females is doubled [48]. Having self-experienced a medication error is another predictor of anxiety revealed by this study: participants who had faced medication errors had higher anxiety scores as compared to participants with no previous experience of these. Experiencing these errors may thus lessen patients’ trust and confidence in their healthcare providers and in the healthcare system in general [31]. According to a study by Nau and Erickson, individuals with experience of medication errors had lower expectations about the safety of the drug dispensing process compared to those without such experience [8]. Similarly, Americans who experienced medical errors were more worried about experiencing them again in the future than those who had never had that experience [42]. Conversely, participants with a medical background were less anxious about being victims of medication errors, perhaps due to their presumed capacity to detect and avert medication errors promptly, as well as their improved understanding that not all such errors result in harm. Moreover, such individuals tend to possess greater assurance in term of managing medication errors, recognising their responsibility to help prevent them, even as a patient.

Participant ability to identify medication errors was also measured in this study, and most participants were able to recognise medication errors committed by doctors or pharmacists, such as prescribing errors (wrong drug, or drug addition or omission) and dispensing errors. Again, this may be because these are the errors that are most frequently encountered [9]. Additionally, more than half of the respondents were able to distinguish adverse drug events from medication errors. Interestingly, however, all participants were least knowledgeable about medication errors committed by patients themselves. Only few of participants knew that incorrect administration or storage of the drug by patients or their caregivers is considered medication error. This suggests that Jordanians are not highly aware of the patient’s role in patient safety. In contrast, a report published in 2017 showed that Americans are highly aware that patients are as responsible as other stakeholders for ensuring patient safety [42]. Awareness of patients’ role in their own care and safety should thus be identified, encouraged, and improved in Jordan.

Patients are usually looked on as the victims of errors [49], yet in this study, 38.1% of participants stated that they always or sometimes forgot to take medications, a rate suggestive of high non-adherence. This indicates that these participants are committing medication errors, yet they remain unaware of this, suggesting that patients may be a major cause of medication errors. Previous studies have showed similar and even higher percentages of non-adherence among patients, both in Jordan [50–53] and internationally [50]. More than three-quarters of participants relied on their own memories to administer their drugs correctly, which may be a contributor to the high level of non-adherence observed, as several studies have shown that using mobile applications can improve adherence [54, 55]. Such applications are now easily accessible, user-friendly, and widely available, yet only 13.7% of the participants in this study use mobile applications to remind them to take the necessary drugs. Patients may thus be encouraged to use special mobile apps more frequently to improve adherence.

Another medication error that participants may commit is storing their medication inappropriately. Storing drugs in the kitchen is one of the most common practices among patients, due to the easy access to water or cutlery used to administer drugs [56, 57]. However, patients should keep any drugs in a special cabinet, away from the sink, stove, or other appliances that emit heat, to avoid storage-related medication errors. One-third of the respondents in this study mentioned keeping drugs in the kitchen, and this makes it worth advising patients further about appropriate storage, as heat and humidity may alter drugs’ properties and reduce their effectiveness or safety.

Having a medical background was the strongest predictor of knowledge in this study (*P* < 0.001). This implies that although patients play a key role in ensuring their own safety, medical professionals still hold the biggest responsibility for educating patients about the part they must play by encouraging them to adopt sound safety principles. Additionally, nationwide education schemes should be organised by the Ministry of Health, Jordan Food and Drug Administration, Jordanian Medical Association, and the Jordan Pharmacist Association to enlighten patients, especially those with no medical background, with regard to patient engagement to help guarantee drug safety and prevent medication errors, thus helping ensure the provision of a quality healthcare system in Jordan.

Using a non-probability sampling method and an online-based survey are considered limitations of this study. This might result in poor representation of the target population especially people who have limited use of social media such as people from rural areas and people of older ages. Additionally, the self-reporting, cross-sectional nature of this study might result in recall bias. Therefore, these limitations should be considered in future research.

## Conclusion

Preventing medication errors is a top priority in all healthcare systems. This study supports a more substantial understanding of medication errors in Jordan from a patient perspective, in addition to offering crucial insights into the prevalence of medication errors, how patients perceive these, and the factors that lead to them. It also emphasises the importance of involving patients in the reporting of medication errors. There is rising concern over the underreporting of medication errors, making it essential to prioritise patient education, particularly in terms of their responsibilities with respect to ensuring medication safety and adherence. Achieving this objective will require a range of collaborative efforts among healthcare professionals and policymakers to implement a range of comprehensive education programmes aimed at promoting patient engagement and enhancing medication safety standards, however.

### Electronic supplementary material

Below is the link to the electronic supplementary material.

Supplementary Material 1

## Data Availability

All data supporting the findings of this study are available from the corresponding author upon reasonable request.
