# Supplementary material for: Unveiling the factors influencing public knowledge and behaviours towards medication errors in Jordan: a cross-sectional study
Source: BMC Health Serv Res. 2024 Jul 10;24:798. doi: 10.1186/s12913-024-11230-6 (PMC11238437; doi:10.1186/s12913-024-11230-6)
Supplement: Supplementary file 1 — Supplementary Material 1 [file 12913_2024_11230_MOESM1_ESM.docx]

**Table S1:** The Questionnaire.

| **Sociodemographic Characteristics.** | | | | | | | | | | | | | | |
| --- | --- | --- | --- | --- | --- | --- | --- | --- | --- | --- | --- | --- | --- | --- |
| **Age (in years)** |  | | | | | | | | | | | | | |
| **Sex** | Male | | | | | | Female | | | | | | | |
| **Education level** | School | | Diploma | | | | University | | | | | Postgraduate | | |
| **Do you have a medical background?** | Yes | | | | | | No | | | | | | | |
| **Do you use drugs regularly?** | Yes | | | | | | No | | | | | | | |
| **Allergies** | Yes | | | | | | No | | | | | | | |
| **Medical insurance** | Yes | | | | | | No | | | | | | | |
| **Medication Safety Behaviour and Medication Errors Experience** | | | | | | | | | | | | | | |
| **How do you remember taking your drugs?** | - I depend on my memory - Someone else reminds me - I use phone applications - Other (Please specify) | | | | | | | | | | | | | |
| **How often do you forget to take your medicine?** | Never | | | Rarely | | | | Sometimes | | | | | Always | |
| **Where do you keep your medication?** | Special cabinet | | | | Bedroom | | | | | | Car | | | |
|  | Kitchen | | | | Office | | | | | | Living room | | | |
|  | Bathroom | | | | Refrigerator | | | | | | Other† | | | |
| **Has a family member or friend experienced medication errors?** | Yes | | | | | | No | | | | | | | |
| **Have you ever experienced a medication error?** | Yes | | | | | | No | | | | | | | |
| **Did you report the medication error?** | Yes | | | | | | No | | | | | | | |
| **Severity of medication error** | - Medication errors that caused permanent disability - Major medication errors that needed medical intervention from the physician or pharmacist - Moderate medication error that needed intervention from the physician or pharmacist - Minor medication error that did not need any intervention | | | | | | | | | | | | | |
| **Anxiety Levels** | | | | | | | | | | | | | | |
| **How anxious are you regarding:** | **Extremely** | **Very** | | | | **Moderately** | | | | **A little** | | | | **Not at all** |
| 1. Drug shortage 2. Inability to afford the drug cost 3. Inability to get correct drug information 4. Experiencing adverse drug events 5. Prescribing the wrong drug by the doctor 6. Buying a drug that was inappropriately stored 7. Dispensing the wrong drug by the pharmacist 8. Buying a counterfeit drug 9. Experiencing Drug-Drug interaction | □  □  □  □  □  □  □  □  □ | □  □  □  □  □  □  □  □  □ | | | | □  □  □  □  □  □  □  □  □ | | | | □  □  □  □  □  □  □  □  □ | | | | □  □  □  □  □  □  □  □  □ |
| **Knowledge About Medication Errors** | | | | | | | | | | | | | | |
| **The following is considered a medication error** | | | **Yes** | | | | | | **No** | | | | | |
| 1. Administering the drug inaccurately by the patient 2. Storing drugs inappropriately by the patient 3. Failing to follow up and monitor treatment with the doctor 4. Failing to have an adequate stock of drugs 5. Ordering or dispensing counterfeit drugs 6. Experiencing an allergic reaction to a drug for the first time 7. Experiencing ADEs despite giving the drug correctly 8. Omission of a necessary drug 9. Addition of an unnecessary drug 10. Storing drugs inappropriately before reaching the patients 11. Dispensing the wrong drug by the pharmacist 12. Prescribing the wrong drug by the doctor | | | □  □  □  □  □  □  □  □  □  □  □  □ | | | | | | □  □  □  □  □  □  □  □  □  □  □  □ | | | | | |
| **The following statement is** | **Correct** | | | | | | **Wrong** | | | | | | | |
| 1. All MEs are preventable 2. ADEs always cause harm to the patients 3. MEs always cause harm to the patients 4. The harm that results from MEs is major 5. Not all ADEs are preventable | □  □  □  □  □ | | | | | | □  □  □  □  □ | | | | | | | |
